# Prognostic Significance and Tumor Immune Microenvironment Heterogenicity of m5C RNA Methylation Regulators in Triple-Negative Breast Cancer

**DOI:** 10.3389/fcell.2021.657547

**Published:** 2021-04-13

**Authors:** Zhidong Huang, Junfan Pan, Helin Wang, Xianqiang Du, Yusheng Xu, Zhitang Wang, Debo Chen

**Affiliations:** ^1^Quanzhou First Hospital of Fujian Medical University, Quanzhou, China; ^2^Shengli Clinical Medical College of Fujian Medical University, Fuzhou, China

**Keywords:** triple-negative breast cancer, m5C RNA methylation regulator, *NSUN2*, *NSUN6* prognostic risk signature, tumor immune microenvironment

## Abstract

**Purpose:**

The m5C RNA methylation regulators are closely related to tumor proliferation, occurrence, and metastasis. This study aimed to investigate the gene expression, clinicopathological characteristics, and prognostic value of m5C regulators in triple-negative breast cancer (TNBC) and their correlation with the tumor immune microenvironment (TIM).

**Methods:**

The TNBC data, Luminal BC data and HER2 positive BC data set were obtained from The Cancer Genome Atlas and Gene Expression Omnibus, and 11 m5C RNA methylation regulators were analyzed. Univariate Cox regression and the least absolute shrinkage and selection operator regression models were used to develop a prognostic risk signature. The UALCAN and cBioportal databases were used to analyze the gene characteristics and gene alteration frequency of prognosis-related m5C RNA methylation regulators. Gene set enrichment analysis was used to analyze cellular pathways enriched by prognostic factors. The Tumor Immune Single Cell Hub (TISCH) and Timer online databases were used to explore the relationship between prognosis-related genes and the TIM.

**Results:**

Most of the 11 m5C RNA methylation regulators were differentially expressed in TNBC and normal samples. The prognostic risk signature showed good reliability and an independent prognostic value. Prognosis-related gene mutations were mainly amplified. Concurrently, the NOP2/Sun domain family member 2 (*NSUN2*) upregulation was closely related to spliceosome, RNA degradation, cell cycle signaling pathways, and RNA polymerase. Meanwhile, *NSUN6* downregulation was related to extracellular matrix receptor interaction, metabolism, and cell adhesion. Analysis of the TISCH and Timer databases showed that prognosis-related genes affected the TIM, and the subtypes of immune-infiltrating cells differed between *NSUN2* and *NSUN6*.

**Conclusion:**

Regulatory factors of m5C RNA methylation can predict the clinical prognostic risk of TNBC patients and affect tumor development and the TIM. Thus, they have the potential to be a novel prognostic marker of TNBC, providing clues for understanding the RNA epigenetic modification of TNBC.

## Introduction

Despite advances in treatment, breast cancer remains a leading cause of cancer-related mortality among women worldwide, accounting for 6.6% of all deaths in 2018 (Bray et al., 2018). Triple-negative breast cancer (TNBC) is often more aggressive than other breast cancer subtypes. Unlike the estrogen receptor (ER)-positive and human epidermal growth factor receptor 2 (HER2) positive subtype, the biology of TNBC includes a high proliferation activity, a high degree of immune infiltration, basal-like or mesenchymal phenotypes, and insufficient homologous recombination (Denkert et al., 2017); its characteristics, including the risk factors, molecular and pathological characteristics, disease course, and sensitivity to chemotherapy, are distinct from those of other breast cancer subtypes (Borri and Granaglia, 2020). Although the basic diagnosis and treatment principles of TNBC are similar to those of other breast cancers, TNBC usually has a poor prognosis due to the lack of expression of targeted hormone receptors and HER2 and an insufficient range of treatment options (Borri and Granaglia, 2020; Cai et al., 2020). Therefore, a comprehensive understanding of the molecular mechanism of TNBC would be beneficial for exploring more effective treatment methods (Ding et al., 2020).

Recent studies have presented RNA modification as an emerging mechanism in gene regulation. This reversible post-transcriptional modification is regulated by “writers” (methyltransferases), “readers,” and “erasers” (demethylase) and affects various molecular functions, such as RNA-protein interaction (Liu et al., 2015), RNA stability (Wang et al., 2014; Yang X. et al., 2017), and translation efficiency (Wang et al., 2015; Schumann et al., 2020). Dysregulation of RNA modifications has been linked to several diseases including cancers, such as leukemia (Paris et al., 2019), breast cancer (Marcel et al., 2013), and prostate cancer (Chen et al., 2017).

5-Methylcytosine (m5C), which exists in mRNAs and ncRNAs, is a common RNA modification in humans (Han et al., 2020). Overall, 95,391 m5C sites in the human genome have been reported to date (Tang et al., 2021), identified with different sequencing methods, including bisulfite sequencing, Aza-IP, and miCLIP-Seq. The writers and readers for m5C modification have been well-studied in previous years. The NOP2/Sun domain family member 2 (*NSUN2*) is considered an important methyltransferase of m5C modification in tRNA, abundant non-coding RNAs, and a small number of mRNAs (Hussain et al., 2013; Blanco et al., 2014; Tang et al., 2021). A recent study showed that *NSUN6* mediates site-specific deposition of m5C in mRNA to control the translation quality (Selmi et al., 2021, 6). Although the demethylase of m5C is still unknown, the conversion from m5C modification to other types of modifications (for example, hm5C) has been reported (Chen L. et al., 2019). Abnormal m5C has been recently found to play carcinogenic roles in several cancers. For example, *NSUN2* and YBX1 drive the onset of human urothelial carcinoma of the bladder (UCB) by targeting the m5C methylation site in the 3′ untranslated region of hepatoma-derived growth factor (HDGF) (Chen X. et al., 2019). In gastric cancer, *NSUN2* acts as an oncogene to repress p57^*Kip2*^ in an m5C-dependent manner, which in turn, contributes to the development of cancer (Mei et al., 2020). However, the role of m5C in breast cancer, especially in TNBC with the worst prognosis, is still unclear.

The purpose of this study was to explore the differentially expressed genes in m5C regulators in different subtypes of BC, mainly in TNBC; identify prognostic genes; and initially explore the correlation of these genes with the clinicopathological characteristics, cell signaling pathways, and tumor immune microenvironment (TIM). Toward this goal, we analyzed data on the 11 currently reported m5C regulators in different subtypes of BC, mainly in TNBC and adjacent normal tissues from The Cancer Genome Atlas (TCGA) and Gene Expression Omnibus (GEO) databases.

## Materials and Methods

### Data Source

RNA-seq transcriptome data of 99 TNBC samples, 779 Luminal BC samples, and 152 HER2 positive BC samples and the corresponding clinical information of TNBC were downloaded from the TCGA data portal: National Cancer Institute Genome Data Sharing Website^1^. RNA-seq data of 212 research samples, including 99 TNBC samples and 113 normal breast tissues, and the corresponding clinical information (Table 1) served as the training cohort. For validation, gene expression profiles from the GEO public dataset^2^ were used. Three datasets, namely, GSE38959, GSE45827, and GSE65194, were selected, with a total of 147 samples. Of these, 112 were TNBC samples, and 35 were normal tissue samples. Besides, the independent GEO dataset (GSE58812) with 107 TNBC samples was used as a validation group to evaluate the specificity and sensitivity of the risk signature.

**TABLE 1:** The clinical characteristics of triple negative breast cancer patients in the training cohort.

**Variable**	**Patients**	**Percentage (%)**
**Age (years)**		
≤50	37	36.6
>50	64	63.4
Unknown	0	0.0
**Gender**		
Male	0	0.0
Female	101	100.0
**T stage**		
T1	24	23.8
T2	63	62.4
T3	10	9.9
T4	4	4.0
**N stage**		
N0	63	62.4
N1	25	24.8
N2	11	8.1
N3	2	2.0
Unknown	0	0.0
**M stage**		
M0	85	84.2
M1	1	1.0
Unknown	15	14.9
**Pathological stage**		
I	17	16.8
II	63	62.4
III	17	16.8
IV	1	1.0
Unknown	3	3.0
Total	101	100.0

*T, tumor; N, nodes; M, metastases*

### Identification of Differentially Expressed Genes in the TCGA Database

A total of 11 m5C RNA methylation regulators, including *NSUN2, NSUN3, NSUN4, NSUN5, NSUN6, NSUN7, DNMT2, DNMT3A, DNMT3B, TET2*, and *ALYREF*, were obtained from published literature (Table 2). The factor expression matrix was used as the clinical information of different subtypes of BC samples and normal breast samples in the TCGA database. Then, the R version (4.0.2) of the limma software package^3^ was used to identify the differentially expressed m5C RNA methylation regulators between the tumor group and the normal tissue group. Genes with adjusted P-values of < 0.05 and | log2(FC)| > 1.0 were considered as differentially expressed genes (DEGs). Subsequently, heat maps and violin maps were used to show the differential expression of m5C RNA methylation regulators between the two groups.

**TABLE 2:** The list of the RNA modifying proteins involved in m5C.

**Regulators**	**Type**
m5C	
NSUN2	“writers”
NSUN3	“writers”
NSUN4	“writers”
NSUN5	“writers”
NSUN6	“writers”
NSUN7	“writers”
DNMT1	“writers”
DNMT3A	“writers”
DNMT3B	“writers”
ALYREF	“readers”
TET2	“erasers”

### GEO Database Verification of the Differentially Expressed Genes

First, we integrated all the samples of the three data sets, significantly increasing the number of samples to 147 samples (112 TNBC samples and 35 normal controls). Then, we used the SVA package in the R computing environment to process the batch effect to avoid generating unreliable results. Next, we used the limma package in the R computing environment to analyze the difference in gene expression between tumor tissue and normal tissue (adjusted *P*-value < 0.05 and | log2 (FC)| > 1.0). Subsequently, heat maps and violin maps were generated to show the differential expression of m5C RNA methylation regulators between the two groups.

### Protein-Protein Interaction Network Construction and Correlation Analysis

The Search Tool for the Retrieval of Interacting Genes (STRING) database^4^ was designed to analyze the protein-protein interaction (PPI) information. To evaluate the potential PPI relationship, the previously identified DEGs were mapped to the STRING database. The PPI pairs with a combined score of 0.4 were extracted. Subsequently, the PPI network was visualized using Cytoscape software^5^. It should be noted that nodes with a higher degree of connectivity tend to be more essential in maintaining the stability of the entire network. The R software was used to calculate the degree of each protein node.

### Construction of the Prognostic Risk Scoring Model

To clarify the relationship between the expression of m5C RNA methylation regulators and overall survival (OS), we performed univariate Cox regression analysis. In the LASSO Cox regression algorithm, the optimal penalty parameter lambda and the corresponding coefficient criteria were determined based on the minimum criteria, through 10-fold cross-validation. Thereafter, an ideal prognostic model based on prognosis-related genes was established. The risk score of the predictive model was calculated as follows:

$$R\,i\,s\,k\,s\,c\,o\,r\,e=\sum_{i=1}^{n}\mathrm{Coefi}*\mathrm{xi}$$

where Coef_*i*_ is the coefficient, and x_*i*_ is the relative expression of each selected gene’s z-score conversion. This formula was used to calculate the risk score of each patient in the TCGA database and the GEO dataset (GSE58812).

### Database Analyses of Differentially Expressed Genes and Proteins in Normal and Breast Cancer Tissues

#### UALCAN Database

The UALCAN^6^ database, developed by the TCGA database and the Clinical Proteomic Tumor Analysis Consortium, was used to analyze the differences in the expression of prognosis-related m5C RNA methylation regulators between breast cancer tissues and normal tissues. The analysis was based on clinicopathological parameters, such as the molecular subtypes of breast cancer and tumor histological grade.

#### Human Protein Atlas

The immunohistochemical images in the Human Protein Atlas (HPA) website^7^ show the expressions of *NSUN2* and *NSUN6* proteins in human normal breast tissue and breast cancer tissue.

#### cBioPortal Database

The cBioPortal for Cancer Genomics database^8^ was used to analyze the frequency of gene alterations (including mutations, deletions, copy number gains, and amplifications) in prognostic m5C RNA methylation regulators in breast cancer. All searches were performed according to the online instructions of the cBioPortal.

#### Gene Set Enrichment Analysis

Gene Set Enrichment Analysis (GSEA) was performed in the TNBC cohort to further understand molecular mechanisms of the prognosis-related genes. Gene sets with both a false discovery rate q-val of < 0.25 and a normalized (NOM) p-value of < 0.05 were considered significant. When normalized enrichment score (NES) is a positive value, the gene is positively related to the pathway, and when NES is negative, it means that the gene is negatively related to the pathway.

#### Tumor Immune Single Cell Hub Database

The Tumor Immune Single Cell Hub (TISCH,^9^), an online database focusing on the tumor microenvironment (TME), collected 76 tumor data sets from 27 cancers, including single-cell transcriptome profiles of nearly 2 million cells. In this study, the diverse cell types and cancer types included in the TISCH database were used to systematically investigate the TME (Table 3) heterogeneity.

**TABLE 3:** The tumor microenvironment including immune cells/inflammatory cells, stromal cells, and malignant cells.

Immune cells	Natural Killer Cells (NK)
	B Cells (B)
	CD4 T Cells (CD4+ T)
	CD8 T Cells (CD8+ T)
	Dendritic Cells (DC)
	Monocytes or Macrophages (Mono/Macro)
	Neutrophils
	Mast Cells (Mast) Regulatory T Cells (Treg) Proliferative T cells (Tprolif)
Stromal cells	Endothelial Cells (Endothelial)
	Fibroblasts (Fibroblasts)
Malignant cells	Malignant Cells (Malignant)

#### TIMER Database

The TIMER database^10^ provides six main analysis modules, allowing users to interactively explore the relationship between immune infiltrates and various factors, including gene expression, clinical results, somatic mutations, and somatic copy number changes. In this study, the TIMER database was used to evaluate the correlation between DEGs and the level of immune cell infiltration. Data on six types of immune-infiltrating cells, namely B cells, CD4+ T cells, CD8+ T cells, neutrophils, macrophages, and dendritic cells, were collected from the database and analyzed.

#### Statistical Analysis

All analyses used R v4.0.2 software. The Wilcox test was used to compare the expression levels of 11 m5C regulators in different subtypes of BC samples and normal breast samples in the TCGA and GEO database. The Spearman test was used to identify correlations between m5C regulators. The median risk value was used as a cut-off value to divide patients into high-risk and low-risk groups. The Kaplan-Meier method was used to assess the correlation between high-risk and low-risk groups and overall survival. Univariate and multivariate Cox regression analysis identified whether risk score, age, stage, T, and N can be used as independent prognostic factors. *p*-values < 0.05 were considered statistically different.

## Results

### Expression of m5C RNA Methylation Regulators in Triple-Negative Breast Cancer

**FIGURE 1:**
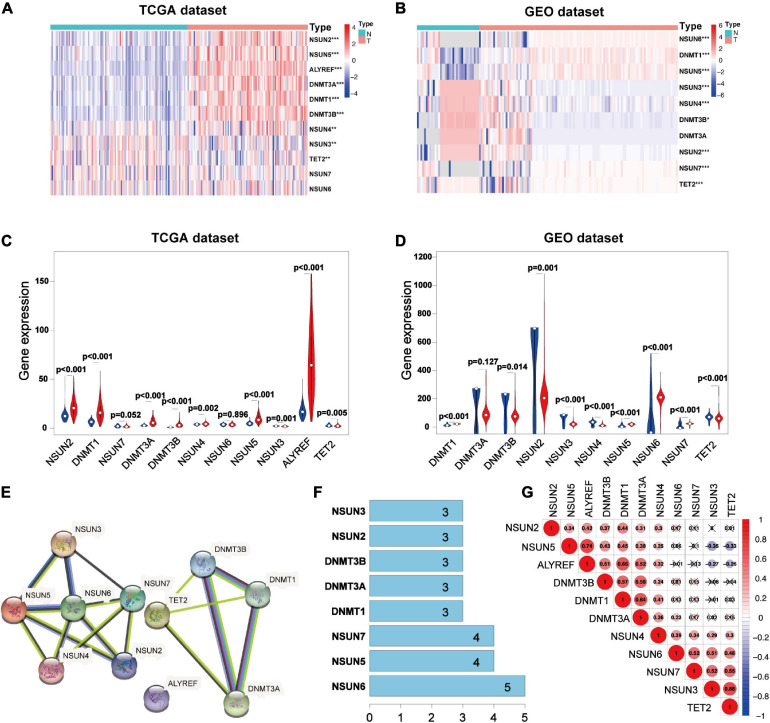
Expression 3D models and correlation of m5C RNA methylation regulators in TNBC. Differential expression heatmap and violin plot of m5C RNA methylation regulators in TNBC and normal tissues from the Human Cancer Gene Atlas **(A,C)** and **(B,D)** Gene Expression Omnibus. The white dot in each “violin” represents the median expression. The PPI network of m5C RNA methylation regulators **(E)**, number of interaction nodes between m5C RNA methylation regulators **(F)**, and Spearman correlation analysis of m5C RNA methylation regulators **(G)**. *p < 0.05, **p < 0.01, and ***p < 0.001. X indicates *p* > 0.05.N, normal sample; T, tumor sample; blue violin: normal sample; red violin: tumor sample; TNBC, triple-negative breast cancer; PPI, protein-protein interaction.

To study the relationship between m5C RNA methylation regulators and BC, we analyzed the expression of m5C RNA methylation regulators in TNBC samples, Luminal BC samples, and HER2 positive BC samples compared with normal tissue samples from the TCGA databases. The TCGA data showed that most of the m5C RNA methylation regulators are abnormally expressed in BC including TNBC (Figures 1A,C and Supplementary Figure 1). In TNBC tissues *NSUN2* (*p* < 0.001), Aly/REF export factor (*ALYREF*, *p* < 0.001), DNA-methyltransferase (*DNMT*)3B (*p* < 0.001), *DNMT1* (*p* < 0.001), *NSUN5* (*p* < 0.001), and *DNMT3A* (*p* < 0.001) were significantly overexpressed than in normal tissues. Meanwhile, *NSUN3* (*p* < 0.005), *NSUN4* (*p* < 0.005), and Tet methylcytosine dioxygenase 2 (*TET2*) (*p* < 0.005) showed significantly lower expression in TNBC tissues. Consistent results were obtained in the analysis of the GEO database.

Analysis of the three data sets of GES38959, GSE45827, and GSE65194 showed that compared with those in normal tissues, the expressions of *DNMT1* (*p* < 0.001), *NSUN5* (*p* < 0.001), *NSUN6* (*p* < 0.001), and *NSUN7* (*p* < 0.001) were significantly higher in TNBC tissues. Meanwhile, the expressions of *NSUN3* (*p* < 0.001), *NSUN4* (*p* < 0.001), *NSUN2* (*p* < 0.001), *TET2* (*p* < 0.001), and *DNMT3B* (*p* < 0.05) were significantly lower in TNBC tissues (Figures 1B,D). Next, we tried to clarify the relationship among the 11 m5C RNA methylation regulators. Analysis of the String database (Figure 1E) showed that *NSUN6* may be a hub gene of the m5C RNA methylation regulator and interacts with the other five genes (Figure 1F). However, further analysis did not show a strong correlation between *NSUN6* expression and the expression of other m5C RNA methylation regulators. Interestingly, *NSUN5* were positively related with four other genes, and its expression had a strong positive correlation with that of *ALYREF* (Figure 1G). *NSUN5* is highly expressed in TNBC, indicating that this gene may be a key gene in the m5C RNA methylation regulator that affects tumorigenesis and development.

### Verification of the Validity of the Risk Signature for Predicting Prognosis

**FIGURE 2:**
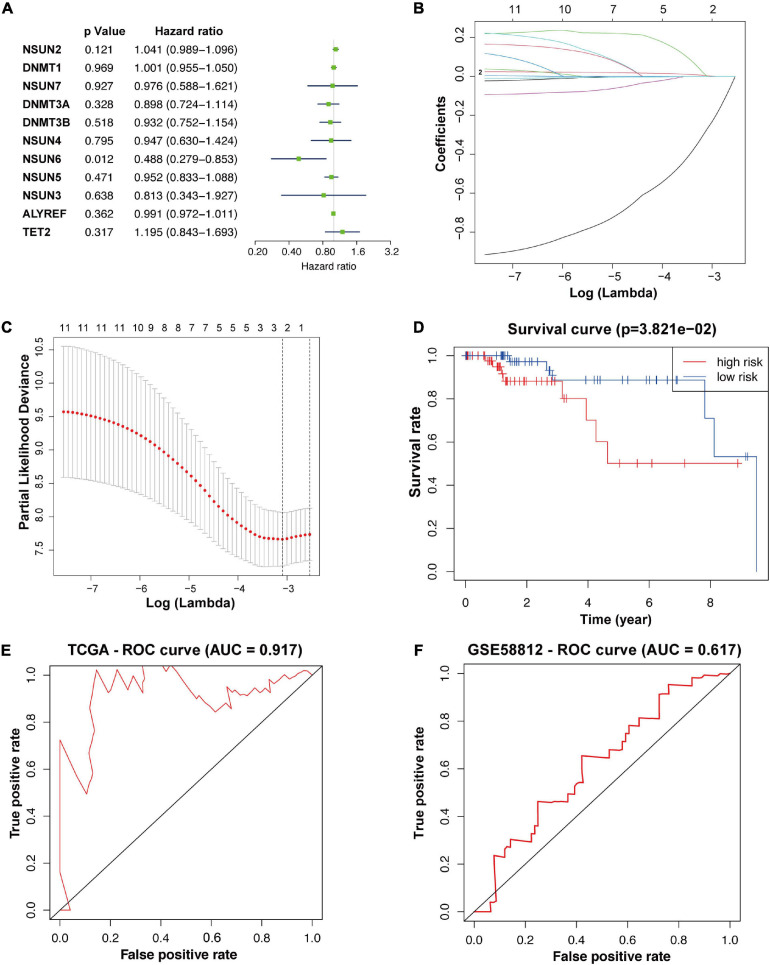
Construction of prognostic risk signature for TNBC patients with two survival-related genes. The *p*-value, HR value, and 95% confidence interval of the 11 m5C RNA methylation regulators analyzed using univariate Cox regression analysis **(A)**. Using LASSO Cox regression, two m5C RNA methylation regulators were selected for risk coefficient calculation **(B,C)**. Kaplan-Meier overall survival curves based on the risk signature **(D)**. ROC curves verified the specificity and sensitivity of the risk signature prediction in the TCGA database and the GEO dataset (GSE58812) **(E,F).** TNBC, triple-negative breast cancer; ROC, operating characteristic curve.

To better understand the prognostic value of m5C RNA methylation regulators in TNBC, we used univariate Cox regression to analyze survival according to the expression of the associated genes in TNBC samples from the TCGA database. The results showed that the two most significant genes influencing OS were *NSUN6* (*p* < 0.05, Figure 2A) and *NSUN2* (*p* < 0.2), with *NSUN6* being a protective factor (hazard ratio, HR < 1) and *NSUN2* being an adverse factor (HR > 1). Then, with expression profiles of eleven m5C RNA methylation regulators, we conducted the LASSO Cox regression algorithm that identified *NSUN2* and *NSUN6*, and constructed a prognostic model based on these two regulators (Figures 2B,C). Lastly, we determined the coefficients used to calculate the risk score after 10-fold cross-validation. The coefficients of *NSUN6* and *NSUN2* were –0.5714 and 0.024, respectively (Table 4). The risk score of each TNBC patient was calculated using the following formula: risk score = −0.5714 × *NSUN6* + 0.024 × *NSUN2*. All TNBC patients were then divided into the low-risk and high-risk groups according to the median risk score. As shown in Figure 2D, patients in the high-risk group had significantly lower OS than those in the low-risk group (*p* < 0.001) (Figure 2D). To evaluate the specificity and sensitivity of the risk signature for predicting TNBC prognosis, we performed a time-dependent receiver operating characteristic curve (ROC) analysis in the TCGA database and the GEO dataset (GSE58812), and the area under the ROC curve (AUC) of the 5-year analysis was 0.917, 0.617, respectively (Figures 2E,F). The above results indicate that our risk signature can predict the prognosis of TNBC.

**TABLE 4:** Genes selected to build risk signature and the corresponding coefficients.

**Genes**	**Coefficients**
NSUN2	0.0248511231827596
NSUN6	−0.571442168838719

### Association Between the Clinicopathological Characteristics and Prognostic Risk Score

**FIGURE 3:**
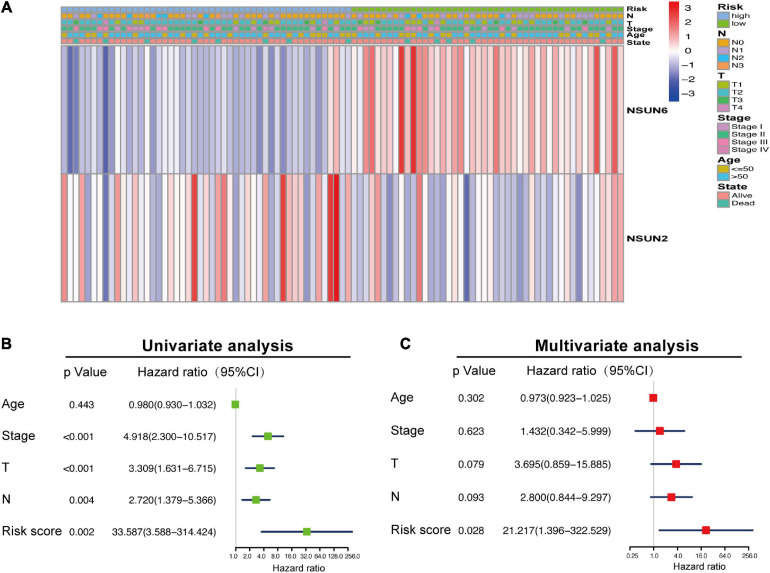
Association between the clinicopathological characteristics and prognostic risk score. The heat map showing the difference in NSUN2 and NSUN6 expression and clinicopathological differences between the high-risk and low-risk groups **(A)**. Univariate and multivariate Cox regression analyses of the association among clinicopathological characteristics, risk score, and overall survival **(B,C)**. NSUN, NOP2/Sun domain family member.

To further verify the prognostic value of the risk signature, we also explored the correlation between the clinicopathological characteristics of TNBC patients and the risk signature. When the data is divided into high-risk and low-risk groups to draw the heatmap, it is regrettable that no significant difference was seen in clinicopathological characteristics (*p* > 0.05) (Figure 3A). Then we performed the univariate and multivariate Cox regression analysis, showed that riskscore had a significant correlation with OS (*p* = 0.002, 0.028, respectively). Meanwhile, univariate Cox regression analysis also showed that TNBC tumor size (T), axillary lymph node metastasis (N), and histological stage were also related to OS (*p* < 0.005) (Figures 3B,C). However, Multivariate Cox regression analysis showed that there was no significant correlation between T, N, M, age, and OS (*p* > 0.05). These results suggest that the risk model established based on the two m5C RNA methylation regulators can be used as an independent prognostic factor for TNBC patients.

### Relationship Between Expression of Prognosis-Related Genes and Clinicopathological Features and Their Protein Expression

**FIGURE 4:**
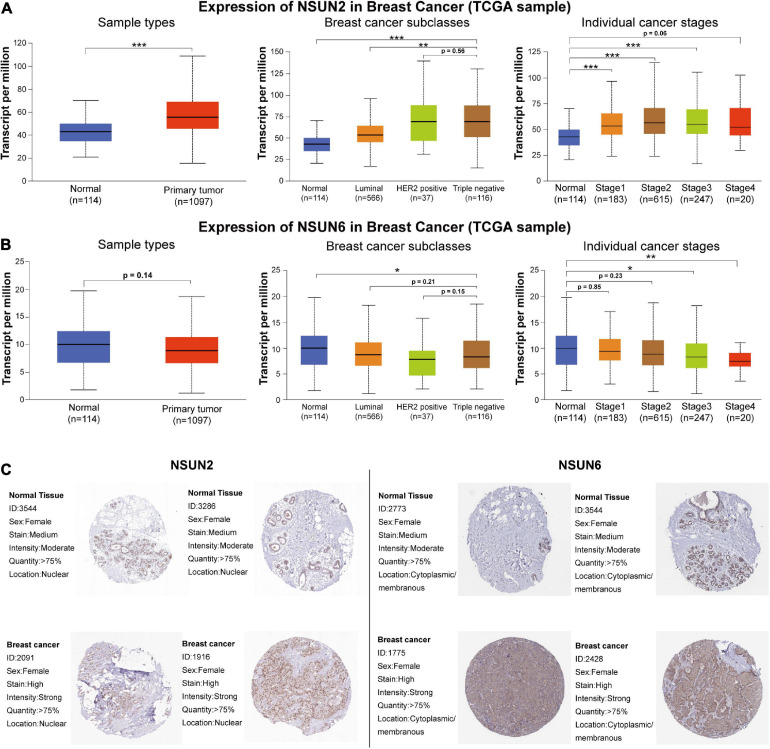
Correlation between the differential expression of the two prognostic genes and clinicopathological patient characteristics and the difference in the expression of these genes at the protein level between normal tissues and breast cancer tissues. mRNA expression of NSUN2 and NSUN6 genes by molecular subtype and histological grade **(A,B)**. The difference in protein expression of NSUN2 and NSUN6 in normal breast tissue and primary breast cancer tissue in the Human Protein Atlas **(C)**. *p < 0.05, **p < 0.01, and ***p < 0.001. NSUN, NOP2/Sun domain family member.

To determine the mechanism by which *NSUN2* and *NSUN6* affected the prognosis of TNBC patients, we used the UALCAN online database to analyze 1,097 primary breast cancer samples and 114 normal breast samples in TCGA according to molecular subtypes and histological classification. The results showed higher *NSUN2* expression in breast cancer tissues than in normal tissues (*p* = 6.38e–09). Interestingly, in the analysis by molecular subtype, *NSUN2* expression was significantly higher in TNBC samples than in both normal samples and luminal breast cancer samples (*p* = 4.12e-10, 1.11e-6, respectively). Meanwhile, there was no significant difference in *NSUN2* expression between TNBC and Her2+ breast cancer (*p* > 0.05). Also, *NSUN2* expression increased in a histology-dependent manner, with a significant difference from level 1 to 3 (*p* < 0.05) (Figure 4A).

For *NSUN6*, there was no significant difference in its expression between normal samples and all primary breast cancers (*p* > 0.05), and no significant in molecular subgroup of breast cancers. In the concurrent analysis by histological classification, *NSUN6* expression was found to be significantly lower in histological grades 3 and 4 TNBC than in normal tissues (*p* < 0.05) (Figure 4B). These findings further confirm the validity of our risk signature, with *NSUN2* as a risk factor and *NSUN6* as a protective factor.

The HPA database was then used to further study the protein expression of *NSUN2* and *NSUN6* at the translation level. The immunohistochemical image showed moderate *NSUN2* expression in normal breast glands and high expression in breast cancer tissues. Meanwhile, *NSUN6* showed medium expression in normal breast glands and high expression in breast cancer tissues (Figure 4C).

### Gene Alterations and Signaling Pathways of Prognosis-Related Genes in Breast Cancer

**FIGURE 5:**
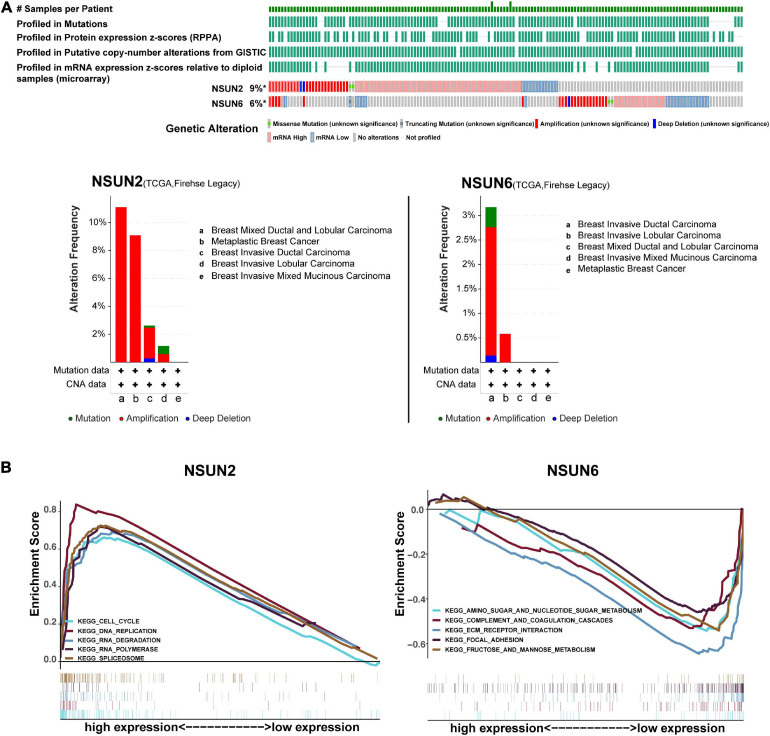
Gene alterations analysis and gene set enrichment analysis of two prognosis-related genes. Analysis of the frequency and distribution of prognosis-related gene alteration in breast cancer based on cBioPortal data **(A)**. GSEA of NSUN2 and NSUN6 in TNBC samples **(B)**. The result is based on NES and NOM *p*-value. GSEA, Gene Set Enrichment Analysis; NSUN, NOP2/Sun domain family member; TNBC, triple-negative breast cancer; NES, normalized enrichment score; NOM, normalized.

Recent studies suggested that gene alterations may affect RNA modifications and disease associations (Song et al., 2020; Chen et al., 2021). Here, we analyzed the alterations in *NSUN2* and *NSUN6* from the cBioportal database, with these alterations mainly including mutations, deletions, copy number gains, and amplifications. The mutation frequencies of *NSUN2* and *NSUN6* were 9% and 6%, respectively, and both were mainly amplified (Figure 5A). We performed GSEA to identify the abnormally activated signaling pathways of *NSUN2* and *NSUN6* that cause their differential expression in breast cancer. Single-gene analysis showed that high *NSUN2* expression was associated with spliceosome (NES = 2.22, *p* < 0.001), RNA degradation (NES = 2.22 *p* < 0.001), cell cycle signaling pathway (NES = 2.12, *p* < 0.001), RNA polymerase (NES = 2.11, *p* < 0.001), and DNA replication (NES = 2.06, *p* < 0.001). Meanwhile, low *NSUN6* expression was associated with extracellular matrix receptor interaction (NES = –1.98, *p* < 0.005), fructose and mannose metabolism (NES = –1.85, *p* < 0.005), amino sugar and nucleotide sugar metabolism (NES = –1.77, *p* < 0.01), complement and coagulation cascades (NES = –1.65, *p* < 0.05), and focal adhesion (NES = −1.64, *p* < 0.05) (Figure 5B).

### Correlation Between the Prognosis-Related Genes and the Tumor Microenvironment

**FIGURE 6:**
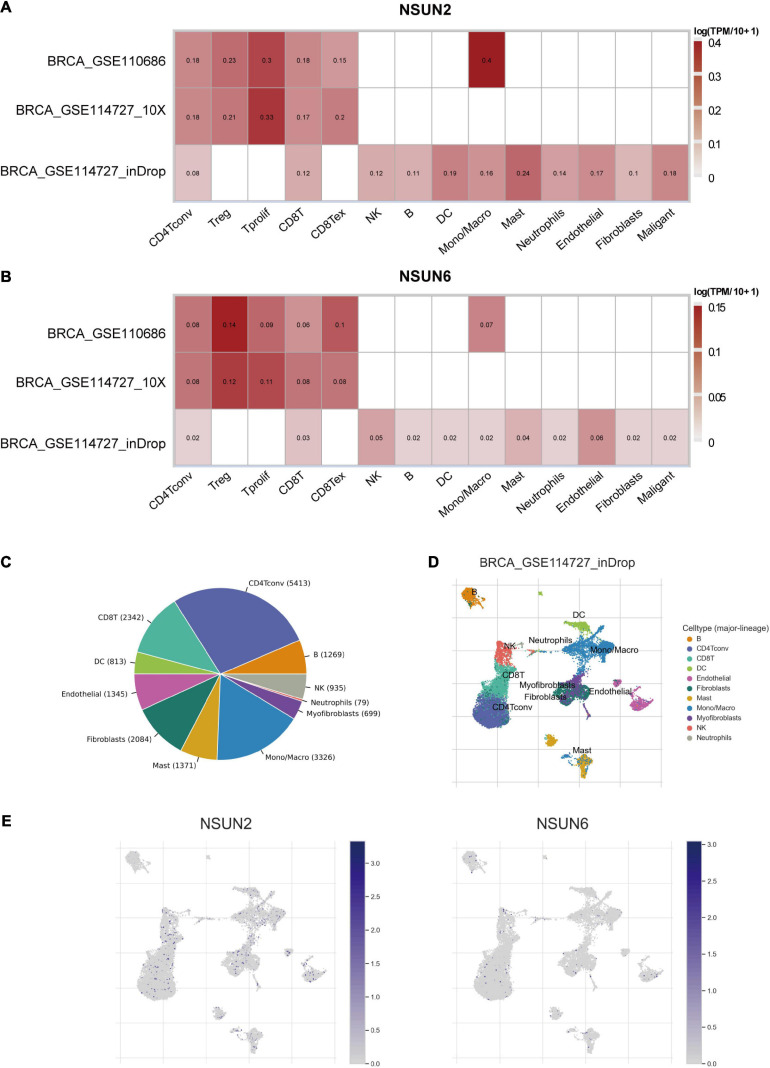
Correlation between the prognostic-related genes and the TME. Correlation analysis between the expressions of NSUN2 and NSUN6 in primary breast cancer tissues and the TME, using the TISCH database **(A,B)**. The cell types and their distribution in the GSE114727_inDrop dataset **(C,D)**. The distribution of NSUN2 and NSUN6 in different cell types was analyzed using single-cell resolution in the GSE114727_inDrop dataset **(E)**. NSUN, NOP2/Sun domain family member; TME; tumor microenvironment; TISCH, Tumor Immune Single Cell Hub.

Considering the role of TME in tumor occurrence and development and its prognostic impact, we used three data sets (i.e., GSE114727-inDrop, BRCA_GSE110686, and BRCA_GSE114727_10X) in the TISCH database to analyze the expression of *NSUN2* and *NSUN6* in TME-related cells. We found low to moderate *NSUN2* and *NSUN6* expression in immune cells including B cells, CD4+ T cells, CD8+ T cells, neutrophils, macrophages, dendritic cells, and Tregs (Figures 6A,B). *NSUN2* expression was the highest in monocytes/macrophages, followed by that in proliferative T cells. Meanwhile, *NSUN6* expression was the highest in Tregs. As shown in the figure, *NSUN2* expression is higher than *NSUN6* expression in immune cells.

We then analyzed the GSE114727-inDrop dataset, which is divided into 12 types of cells. Figure 6C shows the number of cells in each cell type, with the distribution and number of various TME-related cells presented (Figure 6D). In this data set, CD4+ T cells were the most abundant immune cells (*n* = 5413). As shown in Figure 6E, the degree of *NSUN2* infiltration in TME-related cells was higher than that of *NSUN6*, consistent with the results shown in Figure 6A. These results support that m5C regulators are closely related to the TIM in breast cancer.

### Correlation Between Prognostic Genes and TIM

**FIGURE 7:**
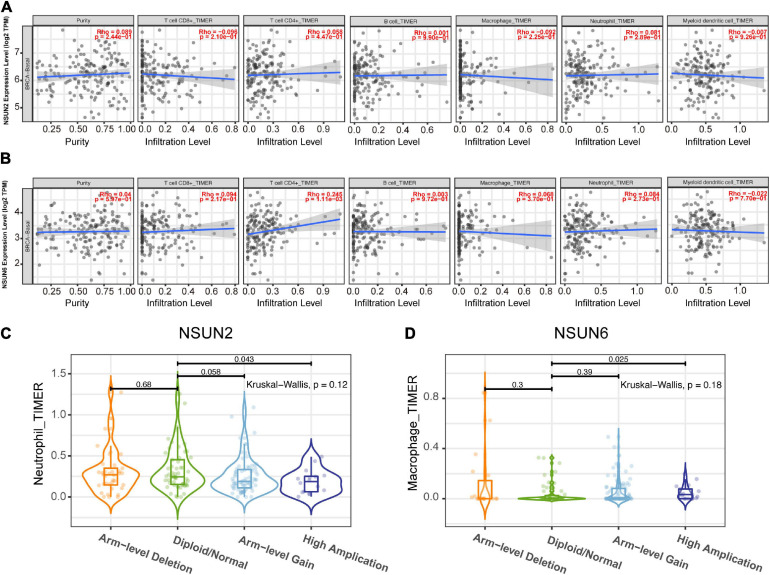
Correlation between prognostic-related genes and TIM. Correlation analysis of NSUN2 and NSUN6 with the infiltration level of the 6 main immune cells after adjusting for the purity **(A,B)**. Analysis according to different groups of somatic copy number alterations showed a significant difference in NSUN2 expression at the neutrophil level **(C)** and NSUN6 expression at the macrophage level **(D)** among these groups. TIM, tumor immune microenvironment; NSUN, NOP2/Sun domain family member.

To determine whether our two prognosis-related genes can reflect the status of tumor immune infiltration, we further used the Timer database to analyze the correlation of *NSUN2* and *NSUN6* with immune cell infiltration in TCGA. As shown in Figure 7A, there was no significant change in *NSUN2* expression (*p* > 0.05) as the degree of immune cell (e.g., CD8 + T cells, CD4 + T cells, B cells, neutrophils, macrophages, and dendritic cells) infiltration increased. However, *NSUN6* was positively correlated with the extent of T cell CD4+ infiltration (*p* < 0.005). Meanwhile, it did not correlate with the other five immune-infiltrating cells (*p* > 0.05) (Figure 7B). However, analysis of the correlation of these two genes with immune cell infiltration showed that under high *NSUN2* amplification, the neutrophil count was significantly lower in samples with somatic copy number alterations than that in normal samples (*p* < 0.05) (Figure 7C). Further, macrophage expression level of *NSUN6* in the high amplification state was higher than that in the normal group (*p* < 0.05) (Figure 7D).

## Discussion

Although RNA modifications have already been reported to be associated with disease pathogenesis and cancer tumorigenesis (Tang et al., 2019; Tian et al., 2020; Zhao et al., 2020), the potential relationships between TNBC and m5C are still unclear. This study found that m5C modification-related regulatory factors were abnormally expressed in TNBC. Concurrently, we found two m5C-modified prognostic genes, and the risk signature constructed based on these two genes showed an independent prognostic value. In addition, these two genes were found to be correlated with the TIM. The value of m5C RNA methylation regulator for predicting the prognosis of TNBC patients and its influence on the TIM of TNBC can be helpful for the diagnosis and treatment of TNBC and ultimately improve patient prognosis. To the best of our knowledge, this is the first study to report the role of m5C RNA methylation regulator in TNBC.

TNBC accounts for approximately 15–20% of all breast cancer cases (Chen et al., 2018), but the standard treatment modality for this subtype of breast cancer lacks effective treatments. Thus, many studies have attempted to develop specific treatment opportunities for TNBC patients in recent years (André and Zielinski, 2012; Garrido-Castro et al., 2019; Yin et al., 2020). An m6A modification has been recently found to play an important role in various human cancers, including breast cancer, by regulating the expression of oncogenes. Similarly, another internal RNA modification, that is, m5C modification, has also been shown to play an important role in cancer. However, related studies on TNBC and m5C modification are still lacking. The number of studies focused on investigating the value of the m5C RNA methylation regulator as a predictor of tumor prognosis is increasing. Hypermethylation of m5C mRNA is highly enriched in cancer-related pathways, such as the PI3K-AKT35 and ERK-MAPK36 pathways (Sun et al., 2001; Samatar and Poulikakos, 2014). Also, the m5C transcription factor *NSUN2* and the m5C transcription factor YBX1 were reported to be abnormally elevated in human UCB. The m5C site-dependent mechanism enhances the stability of HDGF mRNA, thereby promoting the pathogenesis of UCB (Chen X. et al., 2019). In this study, analysis of the expression matrix of 11 m5C RNA methylation regulators in TCGA and GEO databases revealed that they are abnormally expressed in TNBC tissues. This indicated that m5C RNA methylation regulators also play an important role in the pathogenesis of breast cancer.

Methylation regulators of m5C RNA have been verified to be closely related to cancer prognosis. This study found that the m5C RNA methylation regulators *NSUN2* and *NSUN6* are correlated with the prognosis of TNBC. Concurrently, the prognostic risk signature developed based on these two genes has been proven to reliably predict patient prognosis. Patients identified to be at high risk according to the signature were found to have poor prognosis. These findings indicate that m5C RNA methylation regulatory factors also have a prognostic predictive value, and thus, have the potential to be novel prognostic indicators for TNBC patients.

Many studies have confirmed that *NSUN2* and *NSUN6* are related to cancer tumorigenesis. *NSUN2* is a nuclear RNA methyltransferase that catalyzes the formation of 5-methylcytosine. It has been shown to increase protein production through different mechanisms, including by promoting mRNA stability, affecting miRNA maturation and mRNA nuclear export, altering gene and lncRNA expression, and improving protein synthesis and translation. The highly expressed *NSUN2* closely interacts with *RPL6* to promote the proliferation and tumorigenesis of gallbladder cancer cells in both *in vitro* and *in vivo* (Gao et al., 2019). In breast cancer, *NSUN2* is a *MYC* target gene, which is closely related to cell growth and proliferation (Frye and Watt, 2006; Frye et al., 2010; Yi et al., 2017). Besides *NSUN2* expression is negatively correlated with the statue of ER and PR, and is correlated with poor prognosis of BC patients (Yi et al., 2017). *NSUN2* also has a potential prognostic value in other cancers, such as lung cancer, ovarian cancer, and head and neck squamous cell carcinoma (Yang J. et al., 2017; Lu et al., 2020). Analysis of the association between clinical pathology and *NSUN2* expression in breast cancer samples in the UALCAN database showed that *NSUN2* was highly expressed in breast cancer. Further, *NSUN2* expression was also higher in TNBC samples than in the luminal subtype samples.

*NSUN6* is a methyltransferase that targets mRNA. A recent research has shown that Methylation regulated by NUSN6 mainly occurs in the 3′UTR near the translation termination site downstream of the stop codon, and mainly affects the RNA- and protein-binding factors that regulate mRNA processing and translation. In the testis, ovary and liver tissues, the high expression of NSUN6 is related to the high fidelity of RNA- and protein-binding factors, and a better survival rate of patients. Besides, in the Genotypic Tissue Expression (GTEx) database, the expression level of NSUN6 mRNA in normal breast tissue ranked 9th among 22 kinds of tissues (Selmi et al., 2021).” In this study, NSUN6 mRNA expression level was higher in TNBC tissues than in normal tissues. Moreover, we unexpectedly found that NSUN6 is closely related to the stage of breast cancer. It is indicated that in TNBC tissues, the high expression of NSUN6 may also promote the high fidelity of RNA- and protein-binding factors that involve in mRNA processing and translation, and affected the survival of TNBC patients. In addition, analysis using the HPA database showed that *NSUN2* and *NSUN6* were also abnormally expressed at the protein level in breast cancer tissues. This further confirms that *NSUN2* and *NSUN6* are related to the tumorigenesis of breast cancer, consistent with the findings of previous studies (Gao et al., 2019; Selmi et al., 2021).

There have been several reports that gene mutations usually cause phenotypic changes, which are in turn closely related to carcinogenesis and aging (Gonzalo et al., 2017; Kaur et al., 2018; Chen et al., 2021). Concurrently, a meta-analysis of studies on copy number amplification has shown that copy number changes affect genes involved in cell cycle regulation, retinoic acid signaling, complement system, and antigen presentation, which may cause cancer (Brown et al., 2016). The analysis of the cBioPortal database in this study showed that *NSUN2* and *NSUN6* have a high frequency of gene alterations in breast cancer patients. The main gene changes in *NSUN2* and *NSUN6* are copy number amplifications. Succeeding GSEA on *NSUN2* and *NSUN6* in this study showed that these two genes may be involved in important biological processes. *NSUN2* upregulation is closely related to the spliceosome, RNA degradation, cell cycle signaling pathways, and RNA polymerase. *BUD31* is a c-MYC synthetic lethal gene in human breast epithelial cells. *BUD31* has been established to be a component of the core spliceosome required for MYC assembly and catalytic activity (Hsu et al., 2015). In other words, the components of the spliceosome are closely related to aggressive MYC-driven cancers and can be used as therapeutic targets.

In addition, nonsense-mediated RNA decay degrades abnormal RNA and part of normal RNA. RNA degradation can affect various events, including cancer tumorigenesis (Goetz and Wilkinson, 2017). Several studies have shown that cell cycle disorders are the primary driving factor for the immortal proliferation of cancer cells (Hydbring et al., 2016). The mechanisms include activation of cyclin-dependent kinases to drive the entry and development of the cell division cycle, repair of DNA damage, and control of cell death. Cyclin upregulation can lead to cell cycle disorders and uncontrolled cell growth (von Bergwelt-Baildon et al., 2011), indicating that cyclins play a vital role in the pathogenesis of cancer. Regarding RNA polymerase, related studies have shown that certain transforming agents can stimulate the expression of pol III-specific transcription factors TFIIIB or TFIIIC2. Meanwhile TFIIIB is bound and activated by several oncogenic proteins (including c-Myc) and also plays a role in cancer development (White, 2004). Collectively, these findings indicate that the *NSUN2* gene may be involved in important biological processes in cancer tumorigenesis.

Meanwhile, *NSUN6* downregulation is associated with ECM receptor interaction, metabolism, and cell adhesion, and these pathways have been confirmed to be related to cancer. For example, Bao et al. (2019) reported that the ECM-receptor interaction signaling pathway may be related to the development of breast cancer. Research by Akella et al. (2019) showed that changes in metabolism and loss of cell energy are considered hallmarks of all cancers. Also, cell adhesion to the ECM has been recently identified to be a key determinant of drug resistance of cancer cells (Eke and Cordes, 2015). In summary, we speculate that *NSUN6* participates in these pathways in TNBC, ultimately affecting patient prognosis.

The TME comprises stromal cells, bioactive molecules secreted by tumors and stromal cells, the extracellular matrix (ECM), and the lymphatic and vascular systems, all of which play key roles in tumorigenesis and metastasis (Wu and Dai, 2017). TME has been recently widely associated with the prognosis of many cancers and lymph and distant metastasis, including in breast cancer (Zarrilli et al., 2020). A study has found that a variety of transcription factors in the microenvironment induce high expression of microRNA-10b (miR-10b) in metastatic breast cancer cells, promoting cancer cell migration, invasion, and metastasis (Ma et al., 2007). Also, transcription regulators (e.g., nuclear factor-kappa B, mitogen-activated protein kinases, and phosphoinositide-3 kinase/protein kinase-B) are found in thyroid cancer, and these promote the proliferation of oncogenes in the TME, which is in turn related to the thyroid cancer subtype (Ferrari et al., 2020). Immune infiltration in the TME is a current research hotspot. Studies have found that tumor-associated macrophages 1 and 2 and regulatory T cell (Treg) lymphocytes affect the prognosis of ER-positive breast cancer (Hammerl et al., 2018).

However, the impact of TIM on the TNBC progression remains unclear. Compared with other breast cancer subtypes, TNBC is more likely to carry tumor-infiltrating lymphocytes (TILs) (Molinero et al., 2019). Loi et al. (2014) found that for every 10% increase in TILs within the tumor, the risk of death was reduced by 27% in TNBC patients. Further, a high TIL count was an important predictor of long-term recurrence, with every 10% increase reducing the relative risk of long-term recurrence by 13%. Besides, TNBC is rich in CD8+ T cells and is associated with a better prognosis. Compared with other breast cancer types, TNBC has up-regulated expression of regulatory T cells (Tregs) carrying the surface marker FOXP3+ on CD4+ T cells, which may inhibit the effects of other immune cells, thereby inhibiting effective immune responses (Loi et al., 2014). The above studies support that TILs are a powerful prognostic indicator in TNBC. In this study, we used the TISCH database to analyze the correlation between m5C RNA methylation regulators and the TME. The results showed that *NSUN2* and *NSUN6* are expressed to a certain extent in immune cells and *NSUN6* expression was higher than *NSUN2* expression. *NSUN2* is mainly expressed in monocytes/macrophages and proliferative T cells, whereas *NSUN6* is mainly expressed in Tregs. Additional TIMER database searchers were used to show the relationship between two genes with TIM. *NSUN2* and *NSUN6* were correlated with the six major immune cells, with *NSUN6* having the strongest correlation with CD4+T cells.

Monocytes can differentiate into macrophages. Previous studies have shown that tumor-associated macrophages are important immunosuppressive cells that contribute to the growth and metastasis of breast cancer (Yang et al., 2013; Mou et al., 2015). Therefore, the poor prognosis of TNBC may, in part, be attributed to the abnormal expression of *NSUN2* and *NSUN6*, which is in turn, is associated with the upregulation of monocytes/macrophages and Tregs in the TIM.

RNA modification has become an increasingly important field in recent years. To better understand the RNA modifications, several bioinformatics studies were conducted to decipher the functions (Chen K. et al., 2019) and disease association of RNA modifications (Li et al., 2019). However, research on the effect of m5C on breast cancer is still lacking. To the best of our knowledge, this is the first study to conduct an in-depth analysis of the role of m5C RNA regulators in TNBC. However, there are still some limitations in this study. First, there are relatively few TNBC samples (*n* = 99) in the TCGA database, and this may lead to biases in subsequent studies on m5C RNA methylation regulators and clinicopathological characteristics. Second, only one TNBC patient had distant metastases, and thus, it was difficult to analyze the correlation between m5C RNA methylation regulators and M stage. Third, although a correlation analysis between differential genes and immune cancer cells can be performed in the TIMER database, the database cannot correlate clinicopathological characteristics with the degree of immune cell infiltration. Moreover, we were not able to use *in vivo* and *in vitro* experiments to further verify our findings. Fourth, the role of TNBC-related signaling pathways remains unclear. Fifth, we didn’t explore the correlation between m5C regulators and prognosis of non-TNBC patients. Sixth, this study had a limited sample size, so more work needs to be done to strengthen and verify the stability of the risk model. Further molecular biology research and experiments, such as with real-time polymerase chain reaction and western blotting are needed. Future research should overcome these problems.

In conclusion, most m5C RNA methylation regulators are abnormally expressed in TNBC. m5C regulatory factors have the value of predicting prognosis. These regulatory factors are closely related to the tumorigenesis of TNBC and affect TIM. Therefore, m5C RNA methylation regulators have the potential to be prognostic markers for TNBC.

## Data Availability Statement

The datasets presented in this study can be found in online repositories. The names of the repository/repositories and accession number(s) can be found in the article/Supplementary Material.

## Author Contributions

DC and ZH designed the study. ZH and JP collected and analyzed the data. HW wrote the manuscript. XD generated the figures and tables. YX checked and polished the language. ZW reviewed the manuscript. All authors contributed to this article and approved the submitted version.

## Conflict of Interest

The authors declare that the research was conducted in the absence of any commercial or financial relationships that could be construed as a potential conflict of interest.

## Footnotes

1 http://cancergenome.nih.gov/

2 https://www.ncbi.nlm.nih.gov/geo/

3 http://www.bioconductor.org/

4 http://string-db.org/

5 www.cytoscape.org/

6 http://ualcan.path.uab.edu

7 https://www.proteinatlas.org

8 www.cbioportal.org

9 http://tisch.comp-genomics.org

10 https://cistrome.shinyapps.io/timer/

## Supplementary Material

Supplementary Figure 1 Differential expression heatmap and violin plot of m5C RNA methylation regulators in Luminal BC, and HER2 positive BC from the Human Cancer Gene Atlas. ^∗^*p* < 0.05, ^∗∗^*p* < 0.01, and ^∗∗∗^*p* < 0.001. Luminal BC, luminal breast cancer; HER2 positive BC, human epidermal growth factor receptor 2 positive breast cancer.
