# Supplementary material for: Prognostic Significance and Tumor Immune Microenvironment Heterogenicity of m5C RNA Methylation Regulators in Triple-Negative Breast Cancer
Source: Front Cell Dev Biol. 2021 Apr 13;9:657547. doi: 10.3389/fcell.2021.657547 (PMC8076743; doi:10.3389/fcell.2021.657547)
Supplement: Supplementary Figure 1 — Differential expression heatmap and violin plot of m5C RNA methylation regulators in Luminal BC, and HER2 positive BC from the Human Cancer Gene Atlas. ∗p < 0.05, ∗∗p < 0.01, and ∗∗∗p < 0.001. Luminal BC, luminal breast cancer; HER2 positive BC, human epidermal growth factor receptor 2 positive breast cancer. [file Table_1.DOCX]

Supplementary Material


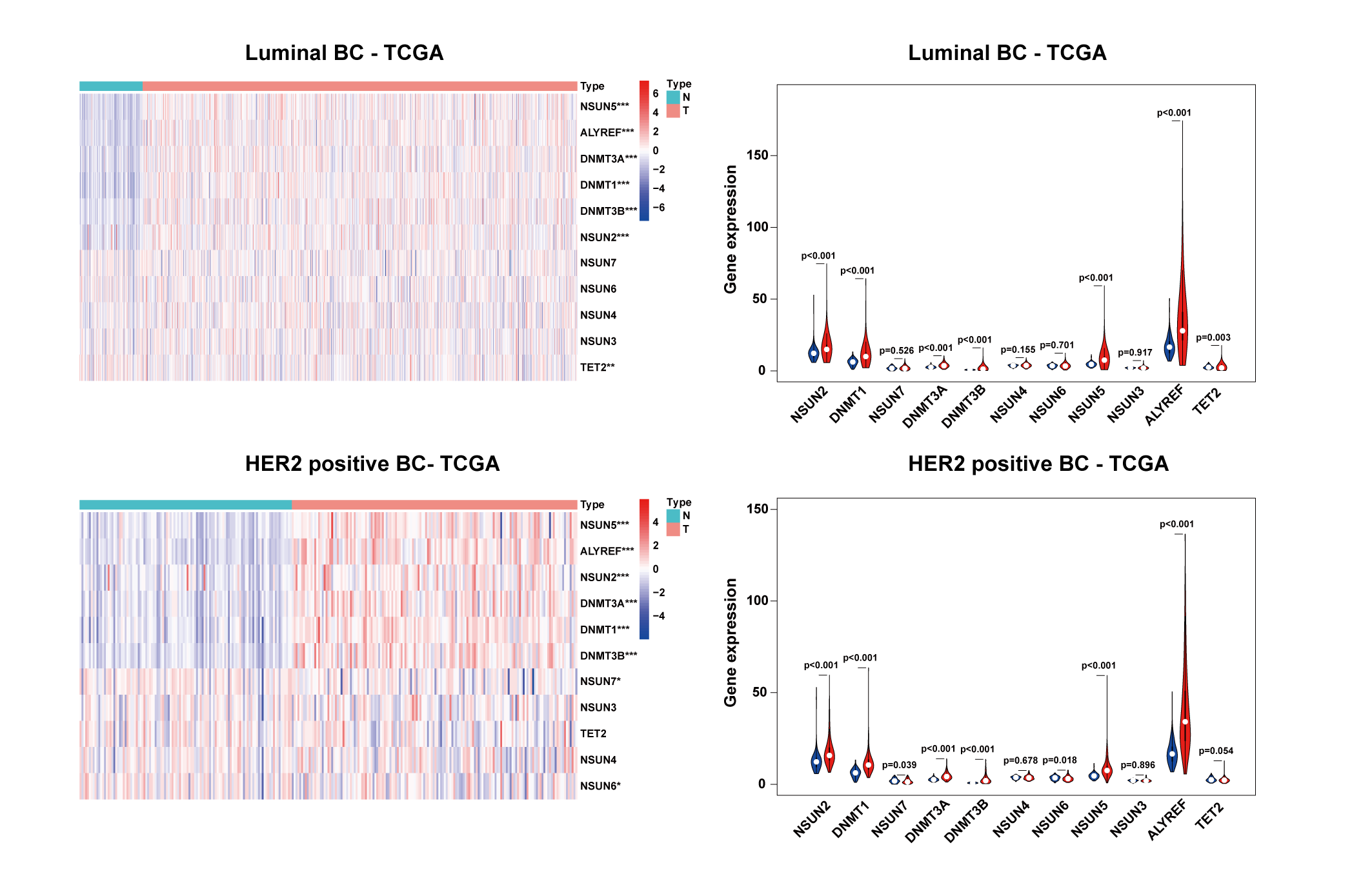


**Supplementary** **Figure 1** Differential expression heatmap and violin plot of m5C RNA methylation regulators in Luminal BC, and HER2 positive BC from the Human Cancer Gene Atlas.

*p < 0.05, **p < 0.01, ***p < 0.001.

Luminal BC, luminal breast cancer; HER2 positive BC, human epidermal growth factor receptor 2 positive breast cancer
